# Efficacy and Safety of Sodium-Glucose Cotransporter-2 Inhibitors in Patients With Refractory Cirrhotic Ascites and Diabetes: Scoping Review Protocol

**DOI:** 10.2196/77587

**Published:** 2026-01-13

**Authors:** Tooba Laeeq, Ayesha Mehak Kamal, Arbab Burhan Uddin Kasi, Umm E Salma Shabbar Banatwala, Aniqa Baloch, Roberto Sagaribay, Vidhani Goel, Aditi Singh, Kavita Batra

**Affiliations:** 1 Department of Internal Medicine, Kirk Kerkorian School of Medicine at UNLV, University of Nevada, 9850 S Maryland PKWY, Las Vegas, NV, 89154, United States, 1 7028950200; 2 Dow University of Health Sciences, New Labour Colony Nanakwara, Karachi, Pakistan; 3 Mass General Brigham, Boston, MA, United States; 4 Office of Research, Kirk Kerkorian School of Medicine at UNLV, University of Nevada, Las Vegas, NV, United States; 5 School of Public Health, University of Nevada, Las Vegas, NV, United States; 6 Department of Medical Education, Kirk Kerkorian School of Medicine at UNLV, University of Nevada, Las Vegas, NV, United States

**Keywords:** sodium-glucose, citransporter-2, diabetes, cirrhotic ascites, diabetes mellitus

## Abstract

**Background:**

Hepatic cirrhosis is a complex condition leading to multiple complications, including ascites, hepatic encephalopathy, bleeding varices, and eventually liver failure. Patients with diabetes mellitus or insulin resistance are more likely to fail treatment, leading to the worsening of hepatic fibrosis. Sodium-glucose cotransporter-2 (SGLT2) inhibitors are a new class of drugs with the potential for use in cirrhotic ascites. This scoping review will focus on the response of the addition of SGLT2 inhibitors (SGLT2i) on refractory ascites.

**Objective:**

The objective of this scoping review is to understand the extent and type of evidence in relation to the response of the addition of SGLT2i on refractory ascites in patients with diabetes mellitus or insulin resistance.

**Methods:**

A comprehensive literature search will be conducted across five databases using variations of the keywords “Sodium-glucose Cotransporter-2 Inhibitors” and “ascites” to identify original studies published from inception through December 2023. The search will be limited to English-language human studies evaluating the efficacy of SGLT2 inhibitors, excluding studies focused on other drugs or conducted in animal models. This review will include studies involving patients over 21 years of age with cirrhotic ascites and will exclude studies involving noncirrhotic causes. Only studies containing original patient data will be considered, regardless of health care setting, to ensure clinical relevance and consistency in interpretation.

**Results:**

The results of this scoping review will offer a comprehensive overview of the available evidence on the use of SGLT2i in the management of cirrhotic ascites. This review will summarize key study characteristics, patient populations, interventions, and outcomes, and identify existing knowledge gaps and research priorities. Findings will be presented narratively and in tabular format to facilitate interpretation and comparison. The review process began with protocol development and registration in February 2025. A structured literature search and database screening will follow, continuing through June. Title and abstract screening, along with full-text review and eligibility assessment, will be completed by July 2025. Data extraction and charting will take place in July, with synthesis and analysis of findings occurring through August. Manuscript drafting will begin in August, and the final review and submission of the completed scoping review are anticipated by September 2025.

**Conclusions:**

By systematically identifying and summarizing original studies that assess the efficacy of SGLT2i in this unique patient population, the review will provide insight into the therapeutic potential of this drug class beyond glycemic control. It will also highlight the strengths and limitations of the existing literature, identify key knowledge gaps, and suggest directions for future research. Ultimately, this review may support the rationale for further clinical investigation and the development of targeted therapies for a high-risk group of patients with limited treatment options.

## Introduction

Liver cirrhosis is a progressive, chronic disease characterized by diffuse hepatic fibrosis and the formation of regenerative nodules, resulting from various etiologies such as viral hepatitis, alcohol-related liver disease, and nonalcoholic steatohepatitis [1-3]. It is a major global health concern, with complications including ascites, hepatic encephalopathy, variceal bleeding, systemic inflammation, and eventual liver failure [2-4]. Ascites, the most common complication, significantly impacts prognosis; approximately half of affected patients die within three years [4]. Globally, cirrhosis-related deaths increased by 8.1% between 2010 and 2019, reaching nearly 1.5 million annually [5], underscoring the urgent need for more effective management strategies.

The standard approach to ascites management includes sodium restriction and diuretic therapy, with large-volume paracentesis reserved for refractory cases [6]. However, these options often become limited due to diuretic resistance, hyponatremia, or renal impairment. An important comorbidity influencing the course of cirrhosis is type 2 diabetes mellitus (T2DM), which is present in roughly one-third of patients and associated with poorer outcomes, including increased risk of ascites development and mortality [7-9]. More than 80% of individuals with cirrhosis have some degree of glucose intolerance [9]. Recent global estimates indicate that T2DM affects approximately 30%‐40% of individuals with cirrhosis, and its presence is associated with a two-fold increase in mortality risk compared to nondiabetic cirrhotic patients [7-9]. This combination not only accelerates hepatic decompensation but also increases the likelihood of developing refractory ascites, underscoring the need for targeted management strategies. Insulin resistance in these patients accelerates hepatic fibrosis and impairs treatment response [10,11].

Selecting safe and effective antihyperglycemic therapy in the setting of chronic liver disease is challenging due to altered drug metabolism and potential hepatotoxicity [12]. While metformin improves insulin sensitivity, conflicting evidence exists regarding its safety in cirrhosis [12-14]. Other agents, such as sulfonylureas and alpha-glucosidase inhibitors, are generally avoided in decompensated cirrhosis, and insulin therapy, though often used, requires further evidence to support its efficacy [12].

Sodium-glucose cotransporter-2 inhibitors (SGLT2i) are antihyperglycemic agents that block renal glucose reabsorption in the proximal tubule, resulting in osmotic diuresis and mild natriuresis [15-17]. These effects may help improve ascites by reducing sodium retention and modulating the renin-angiotensin-aldosterone system [18,19]. Emerging evidence, including preliminary reports in cirrhotic patients with refractory ascites, suggests that SGLT2i may improve ascites management [18,20]. They may also confer additional hepatic benefits, such as improved liver enzyme profiles in patients with coexisting nonalcoholic fatty liver disease [21,22]. However, safety concerns, such as increased risk of genital infections, urinary tract infections, dehydration, acute kidney injury, and euglycemic ketoacidosis, warrant careful patient selection and monitoring [19,23-27].

Despite growing clinical use of SGLT2i in various cardiometabolic conditions, there remains a lack of synthesized evidence specifically evaluating their role in cirrhotic ascites with concomitant diabetes or insulin resistance. No systematic or scoping reviews on this topic were identified in preliminary searches. This scoping review aims to map the extent and type of available evidence regarding the efficacy and safety of SGLT2i in this high-risk population, identify knowledge gaps, and inform the design of future clinical trials. By systematically mapping the current evidence, this review could provide foundational knowledge to guide updates in clinical practice guidelines for managing refractory ascites in patients with cirrhosis and diabetes. Additionally, the synthesis of safety and efficacy data may inform policy decisions regarding the inclusion of SGLT2i as a therapeutic consideration in specific high-risk subgroups, thereby supporting more evidence-based and targeted treatment strategies.

An additional consideration is the issue of diuretic resistance, which frequently complicates the management of refractory ascites. Emerging evidence from heart failure populations suggests that SGLT2i may enhance diuretic responsiveness by promoting natriuresis and improving renal sodium handling [28]. While this mechanism has not been extensively studied in cirrhotic patients, it represents a promising area for investigation and further underscores the potential clinical utility of SGLT2i in the setting of advanced liver disease with fluid overload.

### Objectives

This review aims to examine the impact of SGLT2i on refractory ascites in patients with cirrhosis and coexisting diabetes mellitus or insulin resistance, with a focus on reported safety considerations and adverse events. Additionally, we will assess their effects on clinical outcomes such as glycemic control, fluid status, renal function, and mortality, and explore how their efficacy and safety profile compares to other glucose-lowering agents in this population.

## Methods

### Protocol Registration

The protocol for this scoping review was officially registered on the Open Science Framework (OSF) Registry to ensure transparency, reproducibility, and adherence to established guidelines for systematic reviews. The registration serves as a public record of the review’s objectives, methodology, and planned analysis, allowing for open access to the protocol and facilitating future updates or amendments if needed. The registered protocol is accessible through the following link (10.17605/OSF.IO/7EYCR) [28]. By registering the protocol in this open-access platform, we aim to promote the integrity of the review process and provide a clear framework for the research that can be scrutinized and reproduced by the scientific community.

### Eligibility Criteria

**Figure 1.**
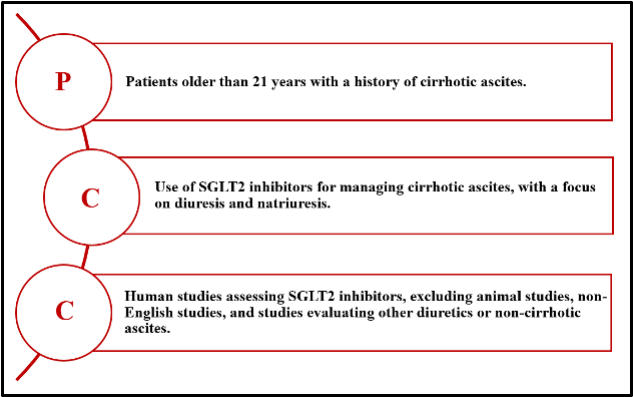
Inclusion criteria based on the participants’ concept context framework. SGLT2: Sodium-glucose cotransporter-2.

The proposed scoping review will follow the PCC framework (Population, Concept, Context) [29] for structuring review questions, identifying relevant literature, and developing inclusion criteria (Figure 1). The results of the search will be reported and presented using Preferred Reporting Items for Systematic Reviews and Meta-analyses extension for scoping review (PRISMA-ScR) guidelines and flow diagram [30,31]. A scoping review design was selected rather than a systematic review because the literature on SGLT2i use in cirrhotic ascites is limited in volume, varied in methodological approach, and often exploratory. Scoping reviews are particularly suited to mapping the extent and nature of research activity, clarifying key concepts, and identifying evidence gaps in emerging fields [29,30]. This approach enables the inclusion of a broader range of study designs, including case reports and small observational studies, which may provide valuable insights for a condition where large-scale randomized trials are scarce.

In terms of participants, patients older than 21 years with a history of cirrhotic ascites will be included. Studies involving patients with ascites due to noncirrhotic causes will be excluded, as will be animal studies, to ensure the findings are relevant to human clinical populations. Regarding the concept, studies evaluating the use of SGLT2i in the management of cirrhotic ascites will be included. Only studies that provide patient data will be included to ensure clinically applicable results. Studies that did not report patient data or primarily assessed diuretics other than SGLT2i, will be excluded to maintain the specificity of the review. In terms of context, this review will consider studies conducted in any health care setting that assess the impact of SGLT2i on cirrhotic ascites. Further, only studies published in English will be included due to the unavailability of translation resources within the review team, which may introduce a risk of language bias.

### Types of Sources

This scoping review will consider both experimental and quasi-experimental study designs, including randomized controlled trials, nonrandomized controlled trials, before and after studies, and interrupted time-series studies. In addition, analytical observational studies including prospective and retrospective cohort studies, case-control studies, and analytical cross-sectional studies will be considered for inclusion. This review will also consider descriptive observational study designs including case series, individual case reports, and descriptive cross-sectional studies for inclusion. Reviews and meta-analyses will be excluded.

### Search Strategy

A comprehensive literature search across five databases (PubMed/Medline, Embase, CINAHL, Cochrane, and Web of Science), using variations of the keywords “Sodium-glucose Cotransporter-2 Inhibitors” and “ascites,” will be used to identify original studies published from inception through December 2023. Results will be limited to human studies published in English (see Table 1 for full inclusion and exclusion criteria). The text words contained in the titles and abstracts of relevant articles, and the index terms used to describe the articles, will be used to develop a full search strategy (see Table 2, data extraction framework). The search strategy, including all identified keywords and index terms, will be adapted for each included database and information source. A draft of the search strategies for each database is provided in Table 3. The reference list of all included sources of evidence will be screened for additional studies.

**Table 1.** Inclusion and exclusion criteria for study selection.

Criteria	Inclusion	Exclusion
Population	Patients older than 21 years with a history of cirrhotic ascites	Patients younger than 21 years or those without cirrhotic ascites
Intervention	Use of SGLT2^a^ inhibitors for treating cirrhotic ascites	Use of diuretics other than SGLT2 inhibitors
Study type	Original human studies with patient data	Animal studies or studies without patient data
Language	Studies published in English	Non-English studies
Condition	Ascites due to cirrhosis	Ascites from other causes

a SGLT2: Sodium-glucose cotransporter-2.

**Table 2.** Data extraction framework.

Extraction criteria	Description
Citation	The authors’ name, title of paper, year published, and journal name
Type of study	General empirical approach
Duration	Length of time patients participated in the study
Sample size (n)	The number of participants in each study
Patient’s age	Mean age of participants in each study
Patient’s gender	Gender breakdown (ie, male/female) for each study
Country	The country where the study was conducted
Cause of cirrhosis	ie, PBC^a^, alcohol, or nonalcoholic steatohepatitis
Type of SGLT2i^b^	ie, empagliflozin, dapagliflozin, or canagliflozin
Comparator	Comparison group that uses a treatment other than SGLT2i
Primary outcome	Primary outcomes found in each study
Secondary outcome	Secondary outcomes found in each study
Key findings	Summary of overall findings and implications
Comparator intervention(s)	Type of comparator reported (eg, insulin, metformin, placebo, diuretic therapy); outcomes summarized descriptively and narratively. No quantitative synthesis or comparative effectiveness analysis planned
Adverse effects	Summary of adverse effects of SGLT2i (eg, genital infections, UTIs^c^, ketoacidosis, hypoglycemia, acute kidney injury, volume depletion); note if severity grading (eg, CTCAE^d^ or study-defined)

a PBC: Primary biliary cholangitis.

b SGLT2i: Sodium-glucose cotransporter-2 inhibitors.

c UTI: urinary tract infection.

d CTCAE: Common Terminology Criteria for Adverse Events.

**Table 3.** Draft search strategies by database.

Database	Search strategy (draft)
PubMed (MEDLINE)	(“Sodium-Glucose Transporter 2 Inhibitors”[Mesh] OR “SGLT2 inhibitors” OR empagliflozin OR dapagliflozin OR canagliflozin OR ertugliflozin) AND (“Ascites”[Mesh] OR ascites OR “fluid retention” OR “refractory ascites”) AND (“Liver Cirrhosis”[Mesh] OR cirrhosis OR “hepatic cirrhosis”)
Embase	(’sodium glucose cotransporter 2 inhibitor’/exp OR ’sglt2 inhibitor’ OR empagliflozin OR dapagliflozin OR canagliflozin OR ertugliflozin) AND ('ascites’/exp OR ascites OR ’fluid retention’ OR ’refractory ascites’) AND ('liver cirrhosis’/exp OR cirrhosis OR ’hepatic cirrhosis’)
Scopus	(TITLE-ABS-KEY (“SGLT2 inhibitors” OR empagliflozin OR dapagliflozin OR canagliflozin OR ertugliflozin) AND TITLE-ABS-KEY (ascites OR “fluid retention” OR “refractory ascites”) AND TITLE-ABS-KEY (cirrhosis OR “hepatic cirrhosis” OR “liver cirrhosis”))
Web of Science	TS= (“SGLT2 inhibitors” OR empagliflozin OR dapagliflozin OR canagliflozin OR ertugliflozin) AND TS= (ascites OR “fluid retention” OR “refractory ascites”) AND TS=(cirrhosis OR “hepatic cirrhosis” OR “liver cirrhosis”)
Cochrane Library	(“SGLT2 inhibitors” OR empagliflozin OR dapagliflozin OR canagliflozin OR ertugliflozin):ti,ab,kw AND (ascites OR “fluid retention” OR “refractory ascites”):ti,ab,kw AND (cirrhosis OR “hepatic cirrhosis” OR “liver cirrhosis”):ti,ab,kw

### Study or Source of Evidence Selection

Following the search, all identified citations will be organized and duplicates removed. Following a pilot test, titles and abstracts will then be screened by two independent reviewers for assessment against the inclusion criteria for the review. Potentially relevant sources will be retrieved in full. The full text of selected citations will be assessed in detail against the inclusion criteria by two independent reviewers. Reasons for exclusion of sources of evidence at full text that do not meet the inclusion criteria will be recorded and reported in the scoping review. Any disagreements that arise between the reviewers at each stage of the selection process will be resolved through discussion or with an additional reviewer. The results of the search and the study inclusion process will be reported in full in the final scoping review.

### Data Extraction and Synthesis

Data will be extracted from all included studies by two independent reviewers using a predefined data extraction framework (see Table 2). The draft extraction tool will first be pilot-tested on a sample of studies to ensure clarity, consistency, and comprehensiveness. Inter-reviewer reliability will be assessed during the pilot phase (eg, via percent agreement), and discrepancies will be discussed and resolved to refine the tool before full data extraction begins. The finalized extraction form may be iteratively modified during the review process, with all modifications documented. Extracted information will include study characteristics, population details, interventions, outcomes (efficacy and safety) and, where relevant, comparator interventions (eg, insulin, metformin, placebo, diuretics). Comparator data will be summarized descriptively and narratively, without formal statistical pooling. Any disagreements during the extraction process will be resolved through discussion or, if needed, by consultation with a third reviewer.

The data extracted from the identified studies will allow us to map existing research outcomes and highlight gaps in the current literature. Primary findings will be systematically organized into overarching themes and sub-themes, including efficacy, safety, and comparator outcomes. Although formal grading of evidence quality will not be undertaken, results will be organized and presented by study design (eg, randomized controlled trials, nonrandomized trials, cohort studies, case-control studies, case series, and case reports) to provide readers with context regarding the methodological rigor of each source.

## Results

The results of this scoping review will provide a comprehensive synthesis of the available evidence regarding the use of SGLT2i in managing cirrhotic ascites. The review will encompass a wide range of studies and will analyze various key elements such as study characteristics, patient demographics, interventions, and clinical outcomes. By focusing on these aspects, the review aims to deliver insights into the effectiveness of SGLT2i in improving refractory ascites in patients with cirrhosis, particularly in those with comorbid diabetes mellitus or insulin resistance.

The review will also highlight the gaps in the current literature, shedding light on areas where further research is needed to optimize treatment strategies and improve patient outcomes. In addition to presenting the findings narratively, a tabular format will be used to systematically summarize the data, facilitating easy comparison across studies and helping identify trends, variations, and patterns in the use of SGLT2i for cirrhotic ascites management.

The anticipated findings will not only contribute to our understanding of the therapeutic potential of SGLT2i in this context but also provide valuable information for clinicians seeking to make evidence-based decisions in managing patients with cirrhosis and ascites. To ensure a structured and efficient review process, the following timeline has been established (as shown in Table 4). This timeline provides a clear and systematic framework for the execution of the scoping review, ensuring that each phase is completed within the designated time frame. Regular progress checks and adjustments will be made as needed to ensure the review is thorough, efficient, and on schedule.

**Table 4.** The suggested timeline of the proposed scoping review.

Milestone	Timeline	Details
Protocol development and registration	February-May 2025	The review protocol was developed and registered on the Open Science Framework (OSF) Registry to ensure transparency and adherence to systematic review guidelines
Structured literature search	June 2025	Comprehensive literature search and database screening across five platforms to identify relevant studies from inception through December 2023
Title and abstract screening	July 2025	Initial screening of identified studies based on title and abstract to determine relevance
Full-text review and eligibility assessment	August 2025	In-depth review of selected studies to assess eligibility according to the inclusion and exclusion criteria
Data extraction and charting	September-November 2025	Systematic extraction of key study data, including patient characteristics, interventions, and outcomes
Synthesis and analysis of findings	December 2025	Analysis of extracted data, synthesis of results, and identification of trends, gaps, and key findings
Manuscript drafting	January-February 2026	Drafting of the manuscript for the scoping review, incorporating the synthesized findings
Final review and submission	March 2026	Final review and submission of the completed scoping review to a peer-reviewed journal

## Discussion

### Principal Findings

This scoping review seeks to provide a comprehensive evaluation of the available evidence regarding the use of SGLT2i in the management of cirrhotic ascites. As cirrhosis and ascites are associated with significant morbidity and limited therapeutic options, particularly in patients with comorbid diabetes or insulin resistance, SGLT2i offer a potentially valuable adjunctive therapy due to their effects on glucose control, renal function, and fluid balance.

Initial studies suggest that SGLT2i may improve fluid status by promoting natriuresis and reducing renal sodium retention, a key mechanism in the pathogenesis of ascites [18]. Moreover, SGLT2i are known to have beneficial effects on glycemic control and may offer an additional advantage in cirrhotic patients who often struggle with diabetes management [32]. However, while their role in diabetes management in cirrhosis is well documented, their specific impact on refractory ascites remains less clear.

The existing body of literature on this topic is still developing, with limited studies specifically addressing the use of SGLT2i in the context of cirrhotic ascites. Early evidence suggests that while these agents may provide therapeutic benefits in terms of fluid balance and glucose control, safety concerns, such as the risk of dehydration, kidney injury, and ketoacidosis, are prevalent in cirrhotic patients, particularly those with advanced liver disease [33]. This highlights the need for careful patient selection and monitoring in clinical practice.

Moreover, the comparative effectiveness of SGLT2i versus other glucose-lowering therapies in cirrhotic patients with ascites has not been fully explored, necessitating future research to establish their relative safety and efficacy. This review will help identify knowledge gaps and will guide future studies, especially randomized controlled trials, to further evaluate the potential benefits and risks of SGLT2i in this complex patient population.

Another potential clinical implication relates to diuretic resistance, which remains a significant challenge in managing refractory ascites. Although direct evidence in cirrhotic populations is limited, studies in heart failure suggest that SGLT2i may enhance diuretic responsiveness through natriuresis and improved renal sodium handling [34]. This raises the possibility that SGLT2i could serve as an adjunctive therapy for patients with cirrhosis and fluid overload who have inadequate responses to conventional diuretics. Future clinical research should explore this mechanism to determine its translational relevance in cirrhotic populations.

### Limitations

Restricting the review to English-language publications may have excluded relevant studies in other languages, introducing potential language bias. The inclusion of diverse study designs, including case reports and small observational studies, may limit the ability to draw firm conclusions about causality or comparative effectiveness. Additionally, as the evidence base is expected to be heterogeneous in methodology and outcome reporting, formal meta-analysis will not be conducted, and findings will be presented narratively, which may limit quantitative synthesis.

### Conclusions

This scoping review will provide a comprehensive overview of the current evidence surrounding the use of SGLT2i in managing cirrhotic ascites, particularly in patients with diabetes or insulin resistance. Although preliminary data suggest that these agents may offer benefits in fluid management and glycemic control, concerns regarding their safety profile in patients with cirrhosis, including potential renal and metabolic risks, warrant further investigation. By identifying key knowledge gaps and highlighting areas requiring additional research, this review aims to contribute to the development of more effective treatment strategies for this challenging and underserved patient population. Further studies, particularly randomized controlled trials, are necessary to confirm the therapeutic role of SGLT2i in cirrhotic ascites and refine treatment protocols.

## Supplementary material

10.2196/77587 Checklist 1 PRISMA-ScR checklist.

## Abbreviations

OSF: Open Science Framework
PRISMA-ScR: Preferred Reporting Items for Systematic Reviews and Meta-analyses-Extension for Scoping Reviews
SGLT2: Sodium-glucose cotransporter-2
SGLT2i: Sodium-glucose cotransporter-2 inhibitors
T2DM: type 2 diabetes mellitus

## References

[1] Sharma B, John S (2023). Hepatic Cirrhosis.

[2] Baumgartner K, Cooper J, Smith A, St Louis J (2021). Liver disease: cirrhosis. FP Essent.

[3] Ginès P, Krag A, Abraldes JG, Solà E, Fabrellas N, Kamath PS (2021). Liver cirrhosis. Lancet.

[4] Pedersen JS, Bendtsen F, Møller S (2015). Management of cirrhotic ascites. Ther Adv Chronic Dis.

[5] Liu YB, Chen MK (2022). Epidemiology of liver cirrhosis and associated complications: current knowledge and future directions. World J Gastroenterol.

[6] Wong F (2023). Innovative approaches to the management of ascites in cirrhosis. JHEP Rep.

[7] Elkrief L, Rautou PE, Sarin S, Valla D, Paradis V, Moreau R (2016). Diabetes mellitus in patients with cirrhosis: clinical implications and management. Liver Int.

[8] Moreau R, Delègue P, Pessione F (2004). Clinical characteristics and outcome of patients with cirrhosis and refractory ascites. Liver Int.

[9] Coman LI, Coman OA, Bădărău IA, Păunescu H, Ciocîrlan M (2021). Association between liver cirrhosis and diabetes mellitus: a review on hepatic outcomes. J Clin Med.

[10] Sapra A, Bhandari P (2023). Diabetes.

[11] Garcia-Compean D, Jaquez-Quintana JO, Gonzalez-Gonzalez JA, Maldonado-Garza H (2009). Liver cirrhosis and diabetes: risk factors, pathophysiology, clinical implications and management. World J Gastroenterol.

[12] Yen FS, Hsu CC, Wei JCC, Hou MC, Hwu CM (2022). Selection and warning of evidence-based antidiabetic medications for patients with chronic liver disease. Front Med (Lausanne).

[13] Yen FS, Huang YH, Hou MC (2022). Metformin use and cirrhotic decompensation in patients with type 2 diabetes and liver cirrhosis. Br J Clin Pharmacol.

[14] Kaplan DE, Serper M, John BV (2021). Effects of metformin exposure on survival in a large national cohort of patients with diabetes and cirrhosis. Clin Gastroenterol Hepatol.

[15] Fonseca-Correa JI, Correa-Rotter R (2021). Sodium-glucose cotransporter 2 inhibitors mechanisms of action: a review. Front Med (Lausanne).

[16] Wright EM (2021). SGLT2 inhibitors: physiology and pharmacology. Kidney360.

[17] Plosker GL (2014). Canagliflozin: a review of its use in patients with type 2 diabetes mellitus. Drugs (Abingdon Engl).

[18] Miyamoto Y, Honda A, Yokose S, Nagata M, Miyamoto J (2023). The effects of SGLT2 inhibitors on liver cirrhosis patients with refractory ascites: a literature review. J Clin Med.

[19] Padda IS, Mahtani AU, Parmar M (2023). Sodium-Glucose Transport Protein 2 (SGLT2) Inhibitors.

[20] Katsiki N, Mikhailidis DP, Theodorakis MJ (2017). Sodium-glucose cotransporter 2 inhibitors (SGLT2i): their role in cardiometabolic risk management. Curr Pharm Des.

[21] Zhao DM, Li CQ, Sun YM (2023). Sodium glucose cotransporter-2-inhibitor dapagliflozin improves nonalcoholic fatty liver disease by ameliorating dipeptidyl-peptidase-4 protein expression in diabetic mice. Endokrynol Pol.

[22] Euh W, Lim S, Kim JW (2021). Sodium-glucose cotransporter-2 inhibitors ameliorate liver enzyme abnormalities in Korean patients with type 2 diabetes mellitus and nonalcoholic fatty liver disease. Front Endocrinol (Lausanne).

[23] D’Andrea E, Wexler DJ, Kim SC, Paik JM, Alt E, Patorno E (2023). Comparing effectiveness and safety of SGLT2 inhibitors vs DPP-4 inhibitors in patients with type 2 diabetes and varying baseline HbA1c levels. JAMA Intern Med.

[24] Khouri C, Cracowski JL, Roustit M (2018). SGLT-2 inhibitors and the risk of lower-limb amputation: Is this a class effect?. Diabetes Obes Metab.

[25] Chen G, Li X, Cui Q (2022). Acute kidney injury following SGLT2 inhibitors among diabetic patients: a pharmacovigilance study. Int Urol Nephrol.

[26] Horii T, Oikawa Y, Kunisada N, Shimada A, Atsuda K (2020). Real-world risk of hypoglycemia-related hospitalization in Japanese patients with type 2 diabetes using SGLT2 inhibitors: a nationwide cohort study. BMJ Open Diab Res Care.

[27] McGill JB, Subramanian S (2019). Safety of sodium-glucose co-transporter 2 inhibitors. Am J Cardiol.

[28] Role of sodium-glucose cotransporter-2 inhibitors in refractory cirrhotic ascites in diabetics – a scoping review. OSF.

[29] Peters MDJ, Godfrey CM, McInerney P, Munn Z, Tricco AC, Khalil H, Aromataris E, Munn Z (2020). JBI Manual for Evidence Synthesis.

[30] Page MJ, McKenzie JE, Bossuyt PM (2021). The PRISMA 2020 statement: an updated guideline for reporting systematic reviews. Syst Rev.

[31] Tricco AC, Lillie E, Zarin W (2018). PRISMA Extension for Scoping Reviews (PRISMA-ScR): Checklist and Explanation. Ann Intern Med.

[32] Siafarikas C, Kapelios CJ, Papatheodoridi M, Vlachogiannakos J, Tentolouris N, Papatheodoridis G (2024). Sodium-glucose linked transporter 2 inhibitors in liver cirrhosis: beyond their antidiabetic use. Liver Int.

[33] Gao FM, Ali AS, Bellomo R (2024). A systematic review and meta-analysis on the safety and efficacy of sodium-glucose cotransporter 2 inhibitor use in hospitalized patients. Diabetes Care.

[34] Stachteas P, Nasoufidou A, Patoulias D (2024). The role of sodium-glucose co-transporter-2 inhibitors on diuretic resistance in heart failure. Int J Mol Sci.

